# Efficient Monitoring of Adult and Immature Mosquitoes Through Metabarcoding of Bulk Samples: A Case Study for Non-Model Culicids With Unique Ecologies

**DOI:** 10.1093/jme/tjaa267

**Published:** 2020-12-10

**Authors:** Pedro M Pedro, Ivy Luizi Rodrigues de Sá, Martha Virginia Ribeiro Rojas, Jandui Almeida Amorim, Allan Kardec Ribeiro Galardo, Noel Fernandes Santos Neto, Nercy Virginia Rabelo Furtado, Dario Pires de Carvalho, Kaio Augusto Nabas Ribeiro, Marcela de Paiva, Maria Tereza Pepe Razzolini, Maria Anice Mureb Sallum

**Affiliations:** 1 Departamento de Epidemiologia, Faculdade de Saúde Pública, Universidade de São Paulo, São Paulo, Brazil; 2 IPE - Institute for Ecological Research, Nazaré Paulista, Brazil; 3 Departamento de Saúde Ambiental, Faculdade de Saúde Pública, Universidade de São Paulo, São Paulo, Brazil; 4 IEPA – Instituto de Pesquisas Cientificas e Tecnológicas do Estado do Amapá, Macapá, Brazil; 5 FUNDUNESP – Fundação para o Desenvolvimento da UNESP, São Paulo, Brazil; 6 Laboratório de Fisiologia e Controle de Artrópodes Vetores do Instituto Oswaldo Cruz- Rio de Janeiro, Brazil; 7 Santo Antônio Energia, Porto Velho, Brazil; 8 Squared Biomonitoring, Nazaré Paulista, Brazil

**Keywords:** *Mansonia* spp, mosquito monitoring, mass-sampling, metabarcoding, ribosomal DNA

## Abstract

The rapid and economical monitoring of mosquitos is imperative to understanding the dynamics of both disease vectors and nuisance species. In light of technological advances in mosquito sampling and DNA sequencing, health agencies can now utilize the full potential of metabarcoding pipelines for rapid and standardizable surveillance. Here, we describe mosquito spatial and temporal variation, with particular focus on *Mansonia* Blanchard species, in the Madeira (Rondônia State) and the Ribeira (São Paulo) watersheds, Brazil using metabarcoding of the D2 rDNA marker. Sampling and molecular pipelines were used to evaluate the taxonomic contribution of mosquitos in pools of culicids collected *en masse* from macrophyte-roots (immatures) and from Mosquito Magnet traps and protected human landings (adults). Results for adult captures are comparable to morphological diagnoses and clarify previously unknown temporal and spatial species turnover. Metabarcoding of immature stages also confirmed the extent of the geographical distribution of some species and each taxon’s association with macrophyte species. Given the benefits of metabarcoding, such as taxonomic acuity, high throughput processing, and objectivity, we suggest such techniques should be more fully incorporated into culicid monitoring schemes. The metabarcoding protocol described herein paired with standardized field sampling schemes, when used by mosquito monitoring professionals, offers substantial improvements in terms of practicality, speed and cost.

Amplicon-based biomonitoring using next-generation DNA sequencing (NGS), known as metabarcoding, is gaining relevance within the mosquito surveillance community (Schneider et al. 2016, Batovska et al. 2018, Pedro et al. 2020). Whereas traditional monitoring is limited by the speed and cost of expert morphological diagnoses, metabarcoding allows for the quick estimate of biodiversity parameters and, by extension, health threats, without the need for taxonomic knowledge in any specific group of mosquitos.

Metabarcoding relies on reference libraries that associate NGS reads to known species (Ratnasingham and Hebert 2007) and can be scaled up to allow for testing hundreds of samples, each containing thousands of mosquitos. However, few of these protocols are both universally applicable across all Culicidae and also practical across sampling schemes, which often rely on captures with substantial non-culicid bycatches. The latter limitation is especially pertinent to adult attractant traps, which are deployed for weeks and capture a substantial quantity of non-culicids (e.g., Pucci et al. 2003).

Immature sampling, particularly in dense aquatic vegetation, will also generate a significant bycatch, which generally must be hand-sorted before downstream analyses (e.g., Sareein et al. 2019). This drawback is especially pronounced for immature collections of *Mansonia* Blanchard species because they attach themselves tenaciously to macrophyte roots and attempts to dislodge them invariably also yield a diverse array of associated aquatic biodiversity (Thomaz et al. 2010).

Although only *Mansonia titillans* (Walker) has been implicated as a pathogen vector in South America (Venezuelan equine encephalitis in Venezuela; Turell 1999), *Mansonia* spp. are nonetheless highly aggressive nuisance species, leading to small-livestock death, large-livestock stress, and pernicious injury to human residents. There is thus a very real need to understand the behavior and ecology of this mosquito taxon, whose temporal and spatial heterogeneity in Brazilian Amazonia has rarely been investigated.

Here we provide the initial results from a nascent metabarcoding surveillance program that tracks macrophyte-associated *Mansonia* Blanchard and other culicid species in the Porto Velho region (Rondônia State) and in southeastern São Paulo State, Brazil. We are undertaking metabarcoding using the D2 ribosomal marker, which was previously shown to out-perform both morphology and mitochondrial markers (including the CO1 barcode) in delineating interspecific mosquito biodiversity. Moreover, because they are fully conserved in the Culicidae, the primers used provide a relatively unbiased copy-number estimate of the D2 in pooled samples (Pedro et al. 2020). The PCR thus recovers accurate relative frequencies of target sites, regardless of which mosquito taxa are represented in the pool.

We are employing D2 metabarcoding for reliable taxonomic diagnoses of captured specimens, which are important to evaluate *Mansonia* species’ preference for different macrophytes (as immatures) and to establish each taxon’s seasonal diversity and habitat partitioning (as immatures and adults). These assessments have immediate practical applications because the main control strategy for *Mansonia* involves the removal of macrophyte beds, which is both an expensive and time-consuming undertaking (Johnstone 1986). Metabarcoding thus provides an efficient means to identify if 1) a given bed hosts species that are of most concern to human and livestock wellbeing and 2) the likely macrophyte breeding habitats that are reservoirs for adult infestations on land. We also assessed *Mansonia* species’ distribution across different biomes (Amazonia and Atlantic Forest) in locations separated by more than 2,000 km (Rondônia and São Paulo states).

## Materials and Methods

### Mosquito Sampling

#### Adults

We used two collection methods: protected human landing and Mosquito Magnet (MM) traps baited with Octenol and Lurex (Table 1). Prior to DNA extraction, the contents of MM traps (samples 77–84 herein) were morphologically identified to genus using the keys of Forattini (2002). Further precision was difficult to achieve because MM specimens were often damaged in the collecting net and lost diagnostic traits. The morphology-based taxonomic assignment of samples 71–76, 244 (human landing) were not available prior to sample homogenization.

**Table 1.** Adult and immature collections used in metabarcoding analyses

ID	Stage	Collection date	Location name	Lat.	Long.	Capture method	Notes
71	Adult	03 May 2019	Sta. Rita (RO)	−9.0331	−64.1485	Human protected landing	~20
72	Adult	29 April 2019	Linha 15 (RO)	−9.0626	−64.4180	Human protected landing	>200 animals
73	Adult	29 April 2019	Linha 17 (RO)	−9.0533	−64.4944	Human protected landing	>100 animals
74	Adult	30 April 2019	São Domingos (RO)	−8.7608	−64.0281	Human protected landing	~20 animals
75	Adult	30 April 2019	Linha 9 (RO)	−8.9773	−64.3186	Human protected landing	>100 animals
76	Adult	02 May 2019	Rio Contra (RO)	−9.3097	−64.4458	Human protected landing	~20 animals
77	Adult	03 June 2019	Jaci Parana (Site 2) (RO)	−9.2633	−64.4050	Mosquito Magnet	~20 animals
78	Adult	03 June 2019	São Domingos (Lot 29-Site 3.1) (RO)	−8.8165	−63.9904	Mosquito Magnet	~100 animals
79	Adult	03 June 2019	Teotonio (Site 1.1) (RO)	−8.8726	−64.0534	Mosquito Magnet	~20 animals
80	Adult	10 June 2019	Teotonio (Site 1) (RO)	−8.8726	−64.0534	Mosquito Magnet	~20 animals
81	Adult	10 June 2019	Teotonio (Site 1.1) (RO)	−8.8726	−64.0534	Mosquito Magnet	~20 animals
82	Adult	03 June 2019	Teotonio (Site 1) (RO)	−8.8726	−64.0534	Mosquito Magnet	~25 animals
83	Adult	10 June 2019	Jaci Parana (Site 2) (RO)	−9.2633	−64.4050	Mosquito Magnet	~50 animals
84	Adult	09 June 2019	São Domingos (Lot 29- Site 3.1) (RO)	−8.8165	−63.9904	Mosquito Magnet	~100 animals
85	Immature	07 June 2019	São Romao 2 (RO)	−9.1855	−64.4292	Agitation and sieving of roots	Collected in Rio Madeira; Macrophyte host: *Eichhornia* Kunth
86	Immature	07 June 2019	São Romao 2 (RO)	−9.1855	−64.4292	Agitation and sieving of roots	Collected in Rio Madeira; Macrophyte host: *Eichhornia* Kunth
87	Immature	07 June 2019	São Romao 2 (RO)	−9.1855	−64.4292	Agitation and sieving of roots	Collected in Rio Madeira; Macrophyte host: *Pistia stratiotes* L.
88	Immature	13 Mar. 2019	Vale do Ribeira (SP)	−24.6026	−47.7595	Agitation and sieving of roots	Collected from puddle in flooded buffalo pasture; Macrophyte host: *Pistia stratiotes* L.
89	Immature	13 Mar. 2019	Vale do Ribeira (SP)	−24.6152	−47.7432	Agitation and sieving of roots	Collected in oxbow lake; Macrophyte host: *Eichhornia* Kunth
90	Immature	13 Mar. 2019	Vale do Ribeira (SP)	−24.6153	−47.7433	Agitation and sieving of roots	Collected in oxbow lake; Macrophyte host: *Eichhornia* Kunth
91	Immature	13 Mar. 2019	Vale do Ribeira (SP)	−24.6154	−47.7432	Agitation and sieving of roots	Collected in oxbow lake; Macrophyte host: *Pistia stratiotes* L.
92	Immature	13 Mar. 2019	Vale do Ribeira (SP)	−24.6154	−47.7432	Agitation and sieving of roots	Collected in oxbow lake; Macrophyte host: *Eichhornia* Kunth
93	Immature	13 Mar. 2019	Vale do Ribeira (SP)	−24.6156	−47.7432	Agitation and sieving of roots	Collected in oxbow lake; Macrophyte host: *Pistia stratiotes* L.
94	Immature	13 Mar. 2019	Vale do Ribeira (SP)	−24.6156	−47.7432	Agitation and sieving of roots	Collected in oxbow lake; Macrophyte host: *Eichhornia* Kunth
95	Immature	13 Mar. 2019	Vale do Ribeira (SP)	−24.6159	−47.7429	Agitation and sieving of roots	Collected in oxbow lake; Macrophyte host: *Pistia stratiotes* L.
244	Adult	21 Aug. 2019	São Domingos (RO)	−8.7608	−64.0281	Human protected landing	~150 animals

RO, Rondônia State; SP, São Paulo State.

#### Immatures

Immature stages of macrophyte-associated *Mansonia* species were sampled, sequenced and assigned to their macrophyte hosts (Table 1). Immatures were collected in both São Paulo and Rondônia states and were all processed as pooled samples with minimal sorting in the field and wet lab. In São Paulo, sample 88 was collected from a puddle in a buffalo pasture that was highly enriched with organic matter. The other seven São Paulo samples (89–95) were collected from an oxbow lake. The Rondônia collections (samples 85–87) were from a highly enriched natural canal adjacent to the Madeira River.

In each collection location, samples were taken from either four *Eichhornia* Kunth (Pontederiaceae) or 10 *Pistia stratiotes* L. (Araceae) roots (Table 1). In both cases, roots were agitated over a white basin for 1 min in the field and discarded back into the water. The contents of the basin were briefly hand-sorted to exclude large remnant roots, leaves, and rocks. However, time was not invested to remove fine roots, other small particles, or invertebrate bycatch. The basin’s contents were filtered and washed twice through a fine mesh (30-count) to remove potentially inhibitory sediment. The collection was then added to a 50 ml falcon tube and topped with 100% ethanol for transport and storage.

A Google Earth (kmz) file is provided identifying immature and adult sampling locations (Supp Data 1 [online only]).

### DNA Extraction From Bulk Samples

All DNA extractions undertaken included negative controls that utilized the same reagents and pipeline (except no tissue was included). This DNA extraction control was included in downstream PCRs to confirm reagents were not contaminated.

#### Adults

The adult extraction protocol used herein can be standardized across pooled samples collected using human landing, MM or other capture methods, such as CDC trapping. It is generally the case that sampling using these protocols does not accumulate appreciable detritus (such as rocks and twigs); however, care was taken to remove unnecessarily burdensome contents such as large leaves, spiderwebs, or very large beetles, whose hard exoskeleton may interfere with the maceration steps.

Because the D2 amplicon used herein is relatively short and the PCR primers are tolerant to bycatch contaminants (Pedro et al. 2020), samples can be used that are moderately degraded and contain substantial non-culicid bycatch. Generally, our MM bycatch included Lepidoptera and spiders, as well as small Diptera and Coleoptera.

The adult pool from both collection methods (including bycatch) was placed into a 50 µl Falcon tube (or more, for larger captures). Samples were dried overnight in the tube(s) using activated silica. Lysis beads were then added (5 ml of 1 mm and 5 ml of 2 mm zirconia beads) and the contents macerated for 30 s using an 850 W jigsaw with a blade adapted to accept the Falcon tube (we used metal hose clamps). Maceration was done with the tube held horizontally.

A 0.1 cm^3^ subsample of the homogenized tissue was transferred into a 2 ml screw-cap lysis tube containing 0.2 mm lysis beads. Tissue replicates were taken as backup samples. Three hundred microliters of distilled water was added to the lysis tube and the tissue further macerated using a Bead Blaster 24 lysis mill (Benchmark Scientific) at maximum rotations for two 10 s cycles.

The homogenate was transferred to new 2 ml Eppendorf tube and a modified salt precipitation was then used to extract DNA. We added 85 µl of 5M NaCl to the 300 µl of the macerated mixture, agitated the tube, and centrifuged for 10 min at room temperature (13,000 g). The supernatant was transferred to a new 1.5 ml Eppendorf tube, 600 µl of absolute ethanol was added and the sample placed on ice for 20 min. The tube was subsequently centrifuged at 4°C for 10 min (13,000 g) and the supernatant discarded. We then added 500 µl of 70% ethanol, inverted the tube to wash salts and spun it at 4°C for 10 min (13,000 g). The 70% ethanol wash was repeated and the supernatant discarded. The pellet was dried for 3 min at 60°C on an Eppendorf Concentrator 5301 vacuum centrifuge. Storage-stock DNA solution was created by the addition of 20 µl of TE buffer and PCR working-stocks used a further 1:20 dilution of the storage-stock.

#### Immatures

Because of the substantial amount of fine plant material present in pooled immature samples collected from macrophyte roots (even after sorting and sieving in the field), we used a CTAB method to economically isolate DNA with minimal amounts of PCR inhibitors. For immature samples taken from less plant-dense habitats, and without substantial detritus, a salt extraction may be used (as above).

Ethanol was removed from the field-collected samples and the contents dried over silica gel (1–2 d). Five milliliters of 1 mm, 5 ml of 2 mm, and 5 ml of 5 mm zirconia lysis beads were then added to the Falcon tube(s) and the contents were macerated as above.

A 0.25 cm^3^ subsample of the macerated tissue was transferred into a 2 ml screw-cap lysis tube containing 0.2 mm lysis beads (replicates were taken as backup samples). Distilled water (300 µl) was added to the lysis tube and the sample macerated using a Bead Blaster 24 as above. The mixture was transferred to new Eppendorf tube and the water evaporated using an Eppendorf Concentrator 5301 vacuum centrifuge.

Five hundred microliters of CTAB extraction buffer (2% CTAB, 1% PVP, 20 mM EDTA, 100 mM Tris HCl, 1.4 M NaCl) was added to the tissue and the mixture was incubated for 30 min at 65°C. The homogenate was centrifuged for 10 min (13,000 × g) and the supernatant transferred to a new 1.5 ml Eppendorf tube. One volume of chloroform/isoamyl alcohol (24:1) was added and the contents vortexed thoroughly. After a 2-min centrifugation (13,000 × g), the upper aqueous phase was transferred to a new tube and 1 ml of absolute ethanol added. Following a 1 h incubation at −20°C, the tube was centrifuged at 13,000 × g for 10 min, the supernatant removed and the pellet washed twice with 500 µl of 70% ethanol. The ethanol was discarded and the tube spun dry in a vacuum centrifuge for 3 min (60°C). The DNA was dissolved in 100 µl of water or TE buffer. This was the working stock used for downstream PCRs.

#### Metabarcoding PCRs

We used the D2 expansion segment as a metabarcoding amplicon target. It has previously been shown to outperform most other markers currently used for mosquito metabarcoding in terms of species resolution and amplifies Culicidae with minimal primer bias.

All PCRs were made to 10 µl and included negative controls for both the original DNA extraction and for the PCR itself. The mixture included final concentrations of: 1× Phusion HF buffer, 0.2 mM dNTPs, 0.2 µM of each Illumina-overhang primer, 0.2U of Phusion High-Fidelity DNA Polymerase (Thermo Fisher Scientific, United States) and one microliter of template DNA. The first-round PCR used forward and reverse primers containing the Illumina overhang sequences (underlined) followed by the sequences of the D2 target: Ill+Mozzie.D2.Uni.F (5′- *TCGTCGGCAGCGTCAGATGTGTATAAGAGACAG* AAGCACTCTGAATAGAGAGTC-3′) and Ill+Mozzie.D2.Uni.R (5′- *GTCTCGTGGGCTCGGAGATGTGTATAAGAGACAG* TGGTCCGTGTTTCAAGAC-3′).

The first-round PCRs used hotstarts initiated at 98°C using an Eppendorf Mastercycler ep Gradient followed by the temperature profiles: 98°C (30 s); 30 cycles of 98°C (10 s), 65°C (30 s), and 72°C (30 s); and a final extension of 72°C for 5 min. Amplicons were visualized on 1% agarose gels and, after confirmation that no primer dimers were present, were diluted 10X prior to the second, indexing, PCR. Here, reagent concentrations were as in the first PCR, except that the concentration of the indexing primers was 0.075 µM. One microliter of the 10X diluted PCR1 product was used as template. Cycling conditions were: initial denaturation at 98°C (10 s); 12 cycles of 98°C (5 s), 55°C (10 s), and 72°C (20 s); and a final extension 72°C for 1 min.

The second PCR products were multiplexed and purified using the QIAquick PCR purification kit (Qiagen, Valencia, CA). The multiplexed mixture was then sequenced using paired-end Illumina MiSeq Reagent v2 Nano chemistry (BPI - Biotecnologia Pesquisa e Inovação, Botucatu, Brazil).

### Sequence Processing and Analyses

We used MOTHUR v.1.36.1 (Schloss et al. 2009) to create contigs from the Illumina paired-end reads using the default *deltaq*=6 as the difference allowed between quality scores of a mismatched base. The *trim.seqs* command was then used to filter contigs with a minimum average quality score of 25 and no nucleotide ambiguities (*maxambig* = 0). Primer sequences were demultiplexed and trimmed using *pcr.seqs* with no mismatched nucleotides allowed in either primer sequence (i.e., *pdiffs* = 0 and *rdiffs* = 0). Clustering of reads into Zero-radius OTUs (ZOTUs) was done using USEARCH v.11.0.667 with the module *unoise3* (Edgar 2010). Here, chimeras were filtered based on *de novo* detection.

We opted for zero-radius OTUs (i.e., all unique sequences were considered separate OTUs), rather than the often-used 3% threshold, because the D2 amplicons contained several indels of varying sizes, even within species. Consequently, single mutational events might lead to inconsistent percent differences between reads and clustering is thus an unreliable indicator of phylogenetic proximity.

We assigned ZOTUs to taxa using the RDP classifier within MOTHUR using *classify.seqs* (Wang et al. 2007) with a D2 database created from all GenBank mosquito entries and our own sequences derived from previous *Mansonia* sequencing efforts (Pedro et al. 2020). RDP, which uses an 8-base subsequence window, allows hierarchical estimates of sequence assignment at various taxonomic levels, in our case from kingdom to species.

Unrooted Neighbor-Joining trees for all mosquito ZOTUs were created with MEGA-X using pairwise deletions and p-distance with bootstrap estimates (Kumar et al. 2016). Additional *Mansonia* spp. D2 sequences from animals previously identified morphologically were included in these trees to assess intraspecific variation and monophyly of all available ZOTUs within species. The Interactive Tree of Life (iTOL) v3 was used for tree editing and annotation (Letunic and Bork 2016).

When evaluating sequence beta diversity among sampling sites, weighted-UNIFRAC distance matrices were created using the PHYLOSEQ R library (McMurdie and Holmes 2013). UNIFRAC is an unbiased metric that is appropriate when molecular species delimitations are not available (as was often the case herein), as it retains the phylogenetic signal of ZOTUs. Confidence values for these were estimated using the *boot.phylo* module within the APEv.5.3 R package (Paradis and Schliep 2019).

We used the VEGAN package (Oksanen et al. 2011) to calculate the Brays-Curtis beta diversity between the MM sampling sites for genera identified by D2 sequences and by morphological diagnoses (a species-level comparison could not be used because of degraded morphological diagnostic traits). Brays-Curtis distance emphasizes both shared taxon identity and abundance and is thus appropriate to test the fidelity of metabarcoding not only in diagnosing presence/absence, but also relative taxon proportions. A Mantel test of the beta diversity matrices was used to confirm the strength of the correlation between sequence- and morphology-based estimates.

## Results

Sequence data for each of the 26 samples analyzed herein are included in NCBI’s Sequence Read Archive under project PRJNA630660 (https://www.ncbi.nlm.nih.gov/sra/) and in Supp Data 2 (online only).

### Adult Culicid Biodiversity

The average number of post-filtering Illumina reads for adult pooled samples was 6,250 (Sequencing depth, which ranged from 1,223 to 36,404, was adjusted for each individual sample prior to multiplexing based on visual estimates of the number of mosquitos in each pool; Table 1). All quality-filtered reads were assigned with high confidence to Culicidae, highlighting the non-amplification of bycatch by the D2 primers. In order to eliminate differences in sampling efficiency between MM and human landing samples, we have analyzed these data separately below.

### Mosquito Magnet

**Fig. 1.**
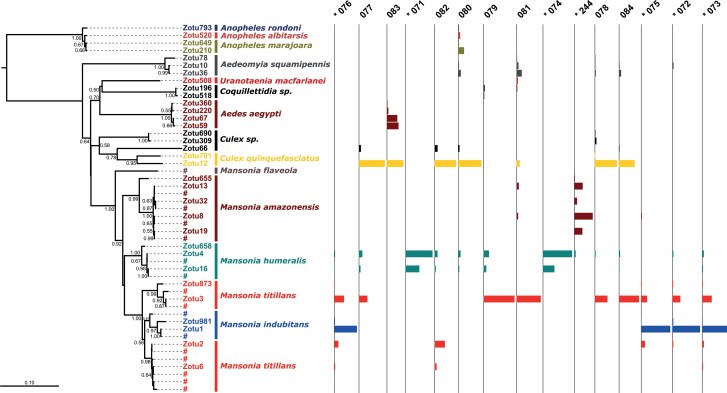
Left: Neighbor-Joining tree (p-distance) estimating the relationship between all ZOTUs identified herein (leaves prefixed with ‘ZOTU’). Leaves prefixed with ‘#’ in the *Mansonia* clade are D2 sequences from specimens previously identified morphologically in Pedro et al. (2020). Bootstrap values above 50% are shown on tree nodes. The taxonomic labels following the ZOTU identity are the lowest level diagnosed by RDP with a confidence value of at least 90. Right: ZOTUs collected at each sampling location. Bar width in each column indicates relative frequency of that ZOTU in the given sample. Samples are ordered to approximate their geographic proximity. Human-landing samples are preceded by an asterisk and Mosquito Magnet samples are without asterisk.

Samples captured using MM traps (77–84; Fig. 1) contained overwhelmingly ZOTUs belonging to *Culex quinquefasciatus* Say and *Mansonia* spp. These two taxa were always sampled in MM except in location 79, which did not contain *Cx. quinquefasciatus* and in location 83, which did not contain *Mansonia*. Sample location 83 contained primarily ZOTUs assigned to *Aedes aegypti* (Linnaeus), a result that we have previously noted using morphological diagnoses in this more urban sampling location. Among the *Mansonia*, MM collections were dominated by *Ma. titillans* and *Ma. humeralis* Dyar and Knab. *Mansonia flaveola* (Coquillett), which we previously sampled in the Porto Velho region of Rondônia, was not among the ZOTUs in the samples analyzed.

Adult D2 metabarcoding generally identified a similar culicid biodiversity as morphological identifications at most sampling locations (Supp Data 3 [online only]). The Mantel test confirmed a significant correlation between beta diversity estimates (*r* = 0.78, *P* = 0.004). There were some instances where taxa that occurred in one dataset did not in the other, but we believe this to be morphological misidentification because many of the MM samples were degraded, making assignment even to genus-level difficult. Moreover, species drop-out during the sequencing pipeline is unlikely, as previous work has shown that D2 sequences are consistently recovered in pools even when they represent as little as 0.2% of the total DNA (Pedro et al. 2020).

**Fig. 2.**
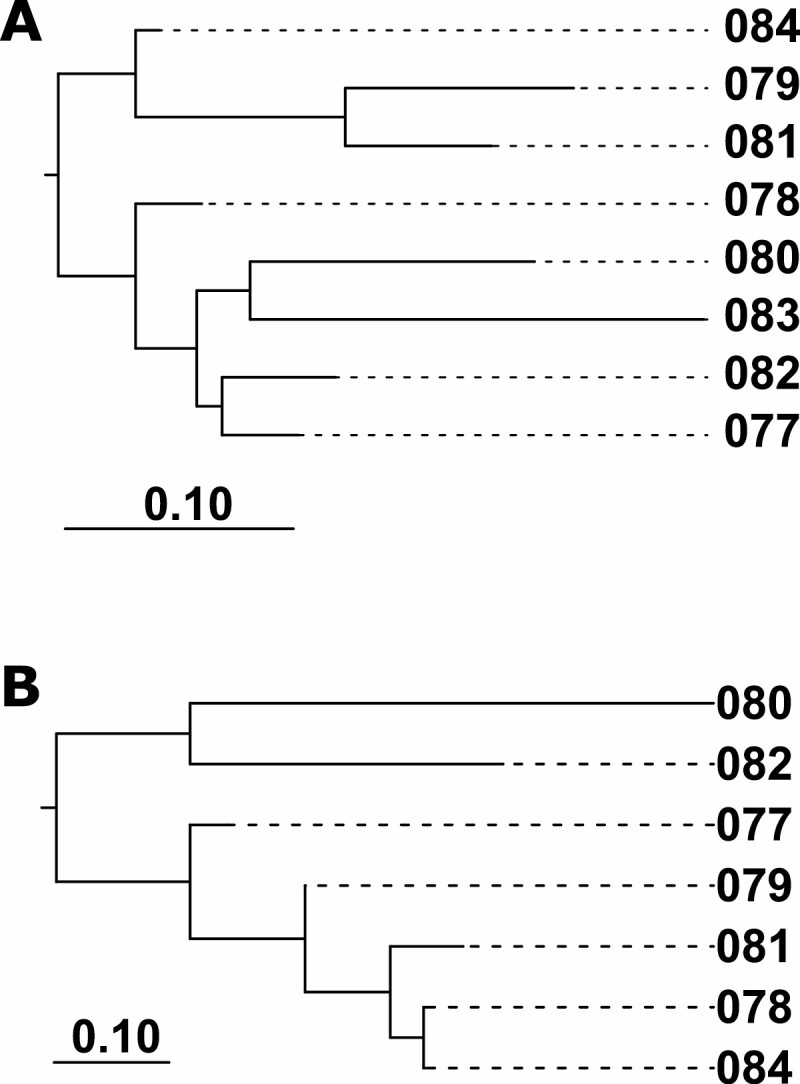
(A) Neighbor-Joining (NJ) tree based on UNIFRAC distances among Mosquito Magnet sampling locations using all Culicidae sequences in samples. (B) NJ tree drawn using only sequences identified as *Mansonia* sp. (sample 83 did not contain *Mansonia* sequences).

When considering all culicid species sequenced from the MM samples, there was a general pattern where pairs with low UNIFRAC distances were also geographically close (Fig. 2A). This is also borne out when evaluating the beta diversity of only *Mansonia* species (Fig. 2B).

### Human Landing

**Fig. 3.**
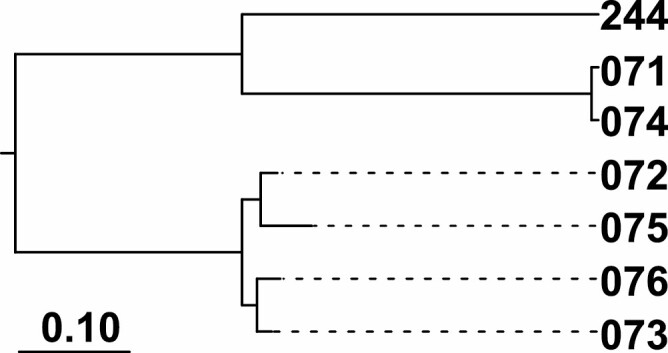
Neighbor-Joining tree of UNIFRAC relationships between adult-landing samples. All nodes had bootstrap values of 100%.

Human landing collections yielded almost exclusively *Mansonia* specimens (samples 71–76 and 244 in Fig. 1), the exception being *Aedeomyia squamipennis* (Lynch Arribalzaga) in sample 72. These few non-*Mansonia* ZOTUs were not considered in the spatial analysis of biodiversity (Fig. 3).

There was a substantial species turnover between wet and dry season samples collected in São Domingos (samples 74 and 244, respectively; Figs. 1 and 3). This changed from almost exclusively *Ma. humeralis* in April to almost exclusively *Ma. amazonensis* (Theobald) in August. There was no apparent differentiation between the left and right banks of the Madeira River, as samples from São Domingos (left bank) and Sta. Rita (right bank) were extremely similar in biodiversity (Fig. 3).

### Immature Culicid Biodiversity

**Fig. 4.**
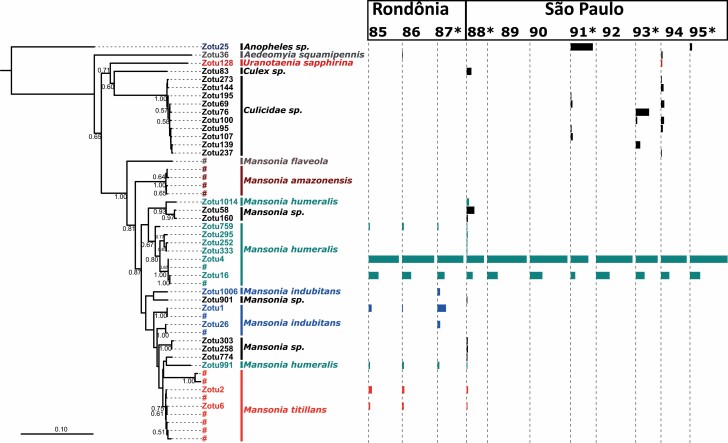
Left: Neighbor-Joining tree of immature-derived ZOTUs identified herein (leaves prefixed with ‘ZOTU’). D2 sequences previously associated with morphologically identified *Mansonia* species (data not shown) are prefixed with ‘#’. Bootstrap values above 50% are shown below branches. RDP identifications are listed after leaf name at the taxonomic level where the confidence is above the 90% threshold. Results are shown for Culicidae only; see Supp Data 4 (online only) for Chironomidae. Right: ZOTUs collected at each sampling location from roots of *Pistia* sp. (location with asterisk) and *Eichhornia* sp. (no asterisk). Bar width indicates relative frequency of that ZOTU in the given sample.

The average number of post-filtering Illumina reads for the 11 immature pooled samples was 10,400 (SD = 3,700). Eighty-six percent of reads were assigned to Culicidae (Fig. 4). The remaining 14% were derived from Chironomidae, as estimated by RDP (Supp Data 2 and 4 [online only]). Thus, unlike in adult samples, some non-culicid aquatic bycatch was also amplified by the molecular pipeline, although this is expected in only a few phylogenetically close Nematocera genera (Pedro et al. 2020). From Ad hoc observations of samples prior to DNA extraction, we estimated that the bycatch in roots was at least three times that of the culicid biomass and included other Diptera immatures, Hemiptera, and aquatic Coleoptera (adult and immature).

**Fig. 5.**
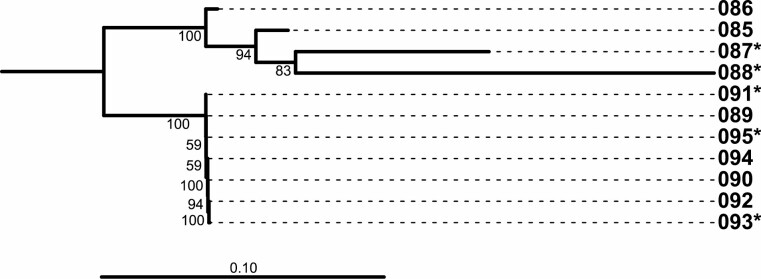
Neighbor Joining tree created using UNIFRAC distances for samples collected from *Pistia* sp. roots (location numbers with asterisk) and *Eichhornia* sp. roots (no asterisk). Samples 85–87 were collected in Rondônia State, the others in São Paulo State. Trees were created using only ZOTUs identified as being from *Mansonia* species (for trees that include other taxa, see Supp Data 5 [online only]).

Macrophytes did not host appreciably different ZOTUs (Fig. 4 and Supp Data 5 [online only]) nor did the results indicate that the two macrophyte species evaluated contained different types of biodiversity, regardless of whether this measure included all Nematocera sequenced, all Culicidae or only *Mansonia* (Fig. 5, Supp Data 5 [online only]). Moreover, ZOTUs belonging to the recognized species *Ma. titillans*, *Ma. indubitans*, and *Ma. humeralis* were found on both macrophytes.

The diversity of *Mansonia* species in Rondônia State was higher than in São Paulo State (Fig. 4). São Paulo samples contained a greater proportion of non-*Mansonia* reads (only one such ZOTU (*Aedeomyia squamipennis*) was found in Rondônia sample 86). In São Paulo, roots sampled from the oxbow river contained only sequences associated with *Mansonia humeralis* (ZOTU 4 and ZOTU 16), which is in agreement with our previous morphological surveys in this area. The buffalo puddle (sample 88) contained ZOTUs assigned to *Ma. titillans* and other, unknown, *Mansonia* D2 clades. Interestingly, sample 88 shared substantial *Mansonia* ZOTU similarity to samples 85–87 (collected in Rondônia State), including ZOTU 759 and sequences phylogenetically close to *Ma. titillans* (ZOTUs 2, 6, and 991; Figs. 4 and 5).

## Discussion

We describe sampling, molecular and analytical protocols for a pilot metabarcoding program that is providing practical monitoring data for the control of culicid species. We undertook PCR on pooled DNA from wild-caught mosquitos using primers that amplify a portion of the Culicidae D2 locus of appropriate size for current Illumina MiSeq chemistry. The PCR, unlike many prior attempts at mosquito metabarcoding, uses primers that are both fully conserved in Culicidae and preclude the amplification of most non-target groups (Pedro et al. 2020). Our protocol is comparably inexpensive and compatible with both freshly collected samples (immatures and landing) and samples from MM and similar traps deployed for 1 wk or longer.

### Adults

In both adult captures (protected human landing and MM), only Culicidae sequences passed read-quality filtering, even when substantial bycatch was present, as in MM samples. The Culicidae-centric primers used are therefore advantageous to programs that use MM and similar traps because these often sample a large quantity of non-culicids, which require substantial sorting efforts in traditional monitoring (Pucci 2003).

Likewise, mass-sampling traps used in monitoring efforts often preclude reliable morphological diagnoses because of damage to samples (Vezenegho et al. 2014). Although we only attained genus-level *morphological* resolution from MM specimens, the resulting *molecular* ZOTUs were all assigned to species-level with high RDP support (Fig. 1), again highlighting the benefits of the molecular approach.

The MM and similar apparatuses are often advantageous alternatives for monitoring efforts because their wider collection windows reduce the potential for outlier samples that can skew the true culicid biodiversity. This is an especially confounding issue in strategies that use punctual sampling, such as human landing or one-night CDC trapping, which yield better-preserved specimens, but are susceptible to biased estimates when captures occur during abnormally cold or wet days (Poh 2019). The efficient quantification of captures using D2 metabarcoding can thus contribute to the wider adoption of these apparatuses.

### Immatures

Although sample bycatch was common to both adult and immature samples, only in the latter did quality-filtered reads include non-Culicidae. These were all assigned to the Chironomidae, which were particularly common in roots from São Paulo State. This co-amplification was not unexpected because chironomids are phylogenetically close to Culicidae and share the 3′ cytosine in the forward D2 primer, which is the main limiter of bycatch amplification (Pedro et al. 2020). Nonetheless, even with the co-amplification of these few non-target taxa, the amount of amplified bycatch is considerably higher when less stringent primers are used, as in Schneider et al. (2016).

Although Rondônia and São Paulo generally grouped together in UNIFRAC analyses (Fig. 5), sample 88 (São Paulo) was associated with Amazonia (Rondônia State) (all were sampled from water highly enriched for organic matter). Given that the ZOTUs shared between sample 88 and Rondônia samples are all phylogenetically proximal to *Ma. titillans* and *Ma. indubitans*, these taxa may be more tolerant of highly turbid conditions.

The *Ma. humeralis* and *Ma. titillans* D2 sequences found in São Paulo State (ZOTUs 4/16 and 2/6, respectively) were identical to those previously found in Rondônia, supporting the conclusion that the same species occur in both locations (Fig. 4; Forattini 2002).

### Additional Considerations

#### Incomplete Species Diagnosis

Several immature *Mansonia* ZOTUs could not be assigned to species with an RDP confidence above 90% (Fig. 4). However, in nearly all these cases, an assignment was estimated that concurred with that ZOTU’s general phylogenetic placement, but with lower confidence. Thus, ZOTUs 58/160 were associated with *Ma. humeralis*, 901/303/258 with *Ma. indubitans*, and 774 with *Ma. titillans*. The low support for these assignments is likely an artifact of an incomplete D2 sequence library (Wang et al. 2007), particularly as our original database was created using only vouchers collected in Rondônia (note that all low-confidence *Mansonia* ZOTUs were collected in São Paulo).

A low-coverage sequence library may also be responsible for the incorrect assignment of ZOTU 991 (Fig. 4), which was assigned by RDP to *Ma. humeralis*, but phylogenetically falls within *Ma. titillans*. Another potential explanation is that this ZOTU represents a previously undescribed species, especially as most immature ZOTUs detected were not found in the adult collections (the reference libraries for both *Ma. humeralis* and *Ma. titillans* were constructed using only adult voucher specimens). This relatively rare read may also be a sequencing error (potentially from chimera formation), although this is unlikely, as the ‘error’ was repeated independently in four samples (85–88).

#### Species Paraphyly

In the adult NJ tree, nearly all species are monophyletic for the D2 locus except for two *Ma. titillans* clades paraphyletic with *Ma. indubitans* (Fig. 1). This supports the existence of a species complex within the *Ma. titillans* morphospecies that we have previously confirmed using the more variable ITS2 marker.

In the immature phylogeny (Fig. 4), the *Mansonia titillans* clade (including the two putative cryptic species) is monophyletic. However, the closely related *Ma. indubitans* (disregarding the ambiguous ZOTU 991) is paraphyletic. This may be a result of at least three dynamics: sequencing error or novel species (as described above) or incomplete lineage sorting in *Ma. indubitans*. Further characterizations of species limits and larger D2 libraries may resolve this ambiguity.

#### Multiple Intragenomic D2 Variants

In Figs. 1 and 4, the number of unique ZOTUs for any one taxon does not always indicate a higher number of individuals for that species, but rather reflect single animals possessing multiple intragenomic variants (Pedro et al. 2020). For example, we previously found that a *Ma. humeralis* individual (when sequenced individually, rather than in a pool) contained multiple D2 copies, the most common of which were ZOTUs 4 and 16 (as in Figs. 1 and 4). Moreover, the respective relative proportions of these two ZOTUs was 3:1 (approximating the results herein). Therefore, the concentration of ZOTUs 4 and 16 should not be used to inflate the number of *Ma. humeralis* individuals in a given sample, but rather be seen as originating from the same individuals. Likewise, the combination of ZOTUs 2 and 6 was also found in *Ma. titillans* specimens sequenced individually, at a concentration of approximately 50:50.

#### Biomass Contribution

Mosquitos vary in size and this variability is broadly correlated to the number of metabarcoding reads for a given species (i.e., the number of cells contributing D2 loci will be greater in larger species). This may initially be an impediment for accurate relative abundance estimates in pools with mixed species. However, repeated sequencing with known numbers of known species will allow for the creation of taxon-specific conversion indices, where the relative frequencies of animals can be extrapolated based on species-specific amplicon contributions (Deagle et al. 2019; Schenk et al. 2019).

Unlike adult captures, immatures will generally be co-sampled at different life stages (Torretta et al. 2006) and thus an equal number of conspecifics may not always contribute the same amount of biomass (and D2 templates) to the PCR. In light of this, relative estimates of immature abundance between species must be carefully assessed. Here, additional sub-sampling from the same site and frequent, repeated, collections may yield the most reliable data.

#### Environmental DNA

The use of Culicidae-targeting primers is relevant to the growing number of biomonitoring initiatives that use aquatic environmental DNA (eDNA), which invariably contains substantial amounts of non-target DNA (Schneider et al. 2016, Boerlijst et al. 2019, Krol et al. 2019). By excluding most of the bycatch from amplification, such a protocol saves both time and financial resources.

### Conclusions

Most of the target taxa, particularly *Ma. titillans* and *Ma. indubitans* are difficult to differentiate morphologically, and even non-degraded adults can be confused (Barbosa et al. 2007). Indeed, the lack of external markers has often necessitated additional characters, such as egg structure (da Silva Ferreira et al. 2020). Here we show that, even from a relatively small number of samples, metabarcoding can identify patterns and produce actionable data for the monitoring and control of morphologically similar mosquito species.

We are using the results to inform future control strategies for *Mansonia* populations, particularly in prioritizing the removal of *Eichhornia* sp. and *Pistia stratiotes* beds, by validating that there is, in fact, no association between mosquito and macrophyte species. Likewise, we are testing the potential that *Ma. titillans* and *Ma. indubitans* prefer aquatic environments with high turbidity.

We are also investigating if the pattern of species turnover between wet and dry seasons seen in samples 74 and 244, where human-landings changed from exclusively *Ma. humeralis* to almost exclusively *Ma. amazonensis* (Fig. 3), are a general pattern in the region.

## Supplementary Data

Supplementary data are available at *Journal of Medical Entomology* online.

Supplemental Data 1: Google Earth kmz file of all collection locations.

Supplemental Data 2: Table describing sequencing results after read filtering.

Supplemental Data 3: Left) Bar graph depicting *morphologically-identified* genera of specimens collected; Right) bar graph depicting DNA-*based identification* of genera using the D2 marker. Samples are ordered according to beta diversity similarity.

Supplemental Data 4: Neighbor-Joining tree of non-Culicidae ZOTUs identified from roots of *Eichhornia* sp. (location numbers with no asterisk) and *Pistia* sp. (asterisk). Although RDP identifications were always a best match to Chironomids, only the taxonomic level where confidence is above the 90% threshold is listed. Bootstrap values above 50% are shown below branches. Bar width indicates relative frequency of that ZOTU in the given sample.

Supplemental Data 5: Neighbor Joining tree created using UNIFRAC distances for samples collected from *Pistia* sp. roots (location numbers with asterisk) and *Eichhornia* sp. roots (no asterisk). Samples 85–87 were collected in Rondônia State, the others in São Paulo State. Trees were created using A) all Nematocera taxa detected in the sequencing output; B) only Culicidae.

tjaa267_suppl_Supplementary_Data_1

tjaa267_suppl_Supplementary_Data_2

tjaa267_suppl_Supplementary_Data_3

tjaa267_suppl_Supplementary_Data_4

tjaa267_suppl_Supplementary_Data_5

tjaa267_suppl_Supplementary_Data_3_Legend

## Data Availability

Sequence data (individual fastq files) are available from the NCBI Sequence Read Archive under accession PRJNA630660 (https://www.ncbi.nlm.nih.gov/sra/).
