# Supplementary figures and images for: Efficient Monitoring of Adult and Immature Mosquitoes Through Metabarcoding of Bulk Samples: A Case Study for Non-Model Culicids With Unique Ecologies
Source: J Med Entomol. 2020 Dec 10;58(3):1210–8. doi: 10.1093/jme/tjaa267 (PMC8122236; doi:10.1093/jme/tjaa267)

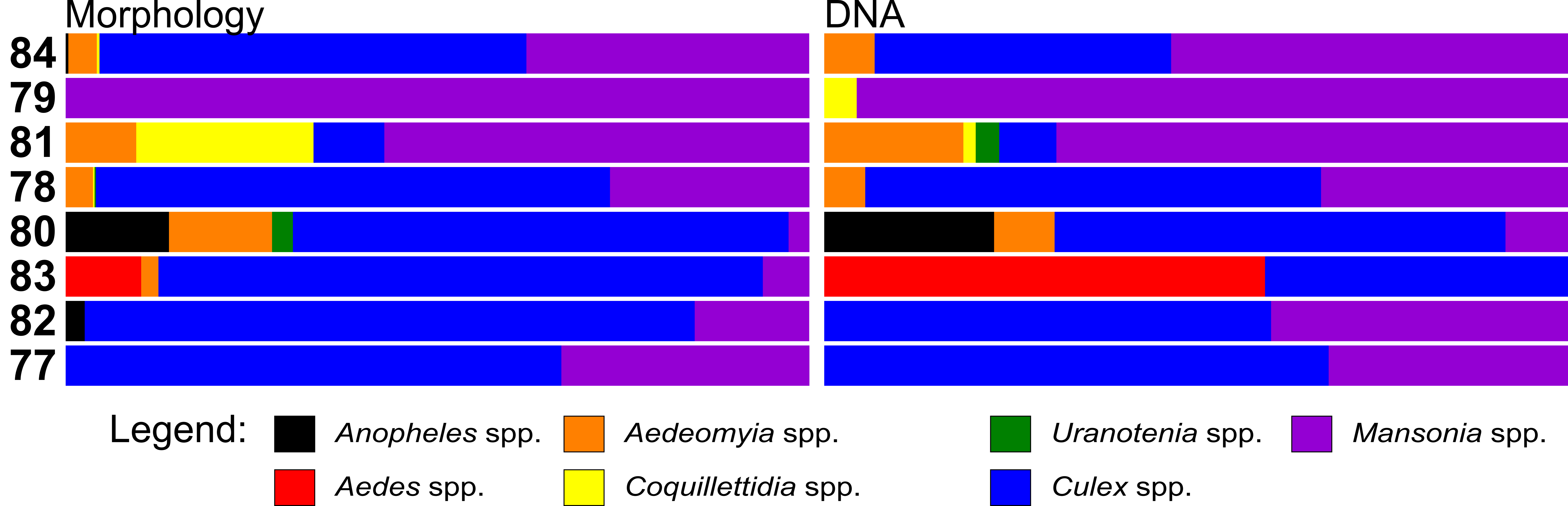

Supplement: tjaa267_suppl_Supplementary_Data_3 [file tjaa267_suppl_supplementary_data_3.pdf]

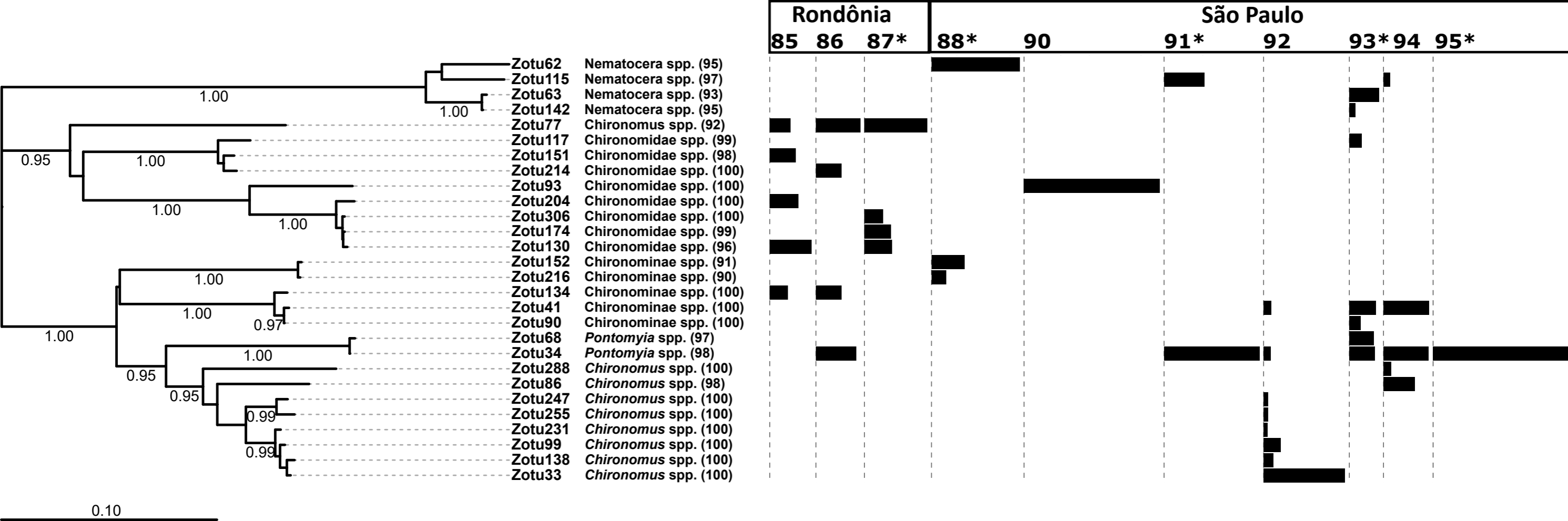

Supplement: tjaa267_suppl_Supplementary_Data_4 [file tjaa267_suppl_supplementary_data_4.pdf]

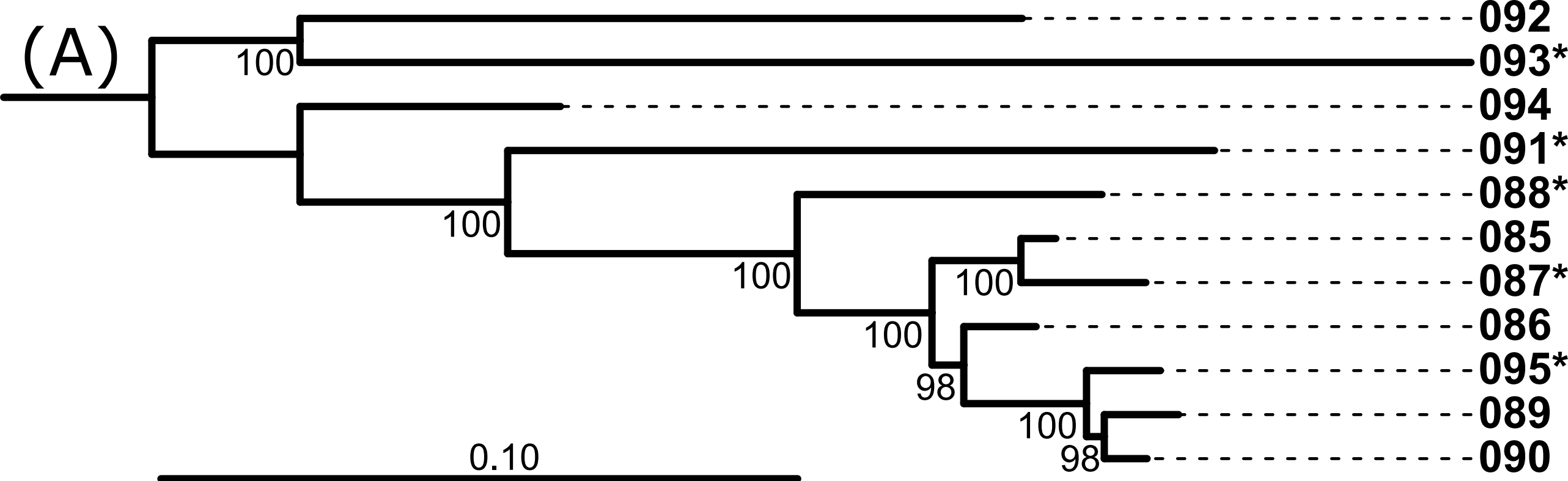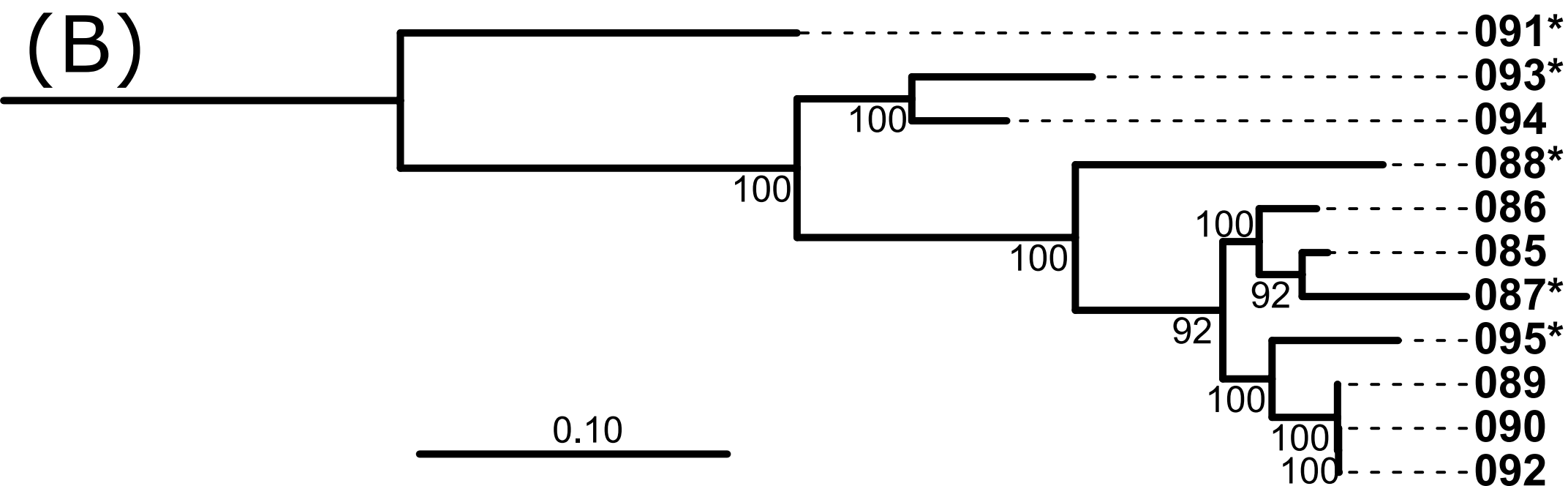

Supplement: tjaa267_suppl_Supplementary_Data_5 [file tjaa267_suppl_supplementary_data_5.pdf]
