# Supplementary material for: Efficient Monitoring of Adult and Immature Mosquitoes Through Metabarcoding of Bulk Samples: A Case Study for Non-Model Culicids With Unique Ecologies
Source: J Med Entomol. 2020 Dec 10;58(3):1210–8. doi: 10.1093/jme/tjaa267 (PMC8122236; doi:10.1093/jme/tjaa267)
Supplement: tjaa267_suppl_Supplementary_Data_3_Legend [file tjaa267_suppl_supplementary_data_3_legend.docx]

Supplemental Data 3: Left) Bar graph depicting *morphologically*-*identified* genera of specimens collected; Right) bar graph depicting *DNA-based* *identification* of genera using the D2 marker. Samples are ordered according to beta diversity similarity.
